# Supplementary material for: Impaired Glycolysis Promotes Alcohol Exposure-Induced Apoptosis in HEI-OC1 Cells via Inhibition of EGFR Signaling
Source: Int J Mol Sci. 2020 Jan 11;21(2):476. doi: 10.3390/ijms21020476 (PMC7014033; doi:10.3390/ijms21020476)
Supplement: Supplementary file 1 [file ijms-21-00476-s001.pdf]

Supplemental information

Impaired glycolysis promotes alcohol exposure-induced apoptosis in HEI-OC1 cells via inhibition of EGFR signaling.

[§] Hyunsook Kang [1], [§] Seong Jun Choi [1], Kye Hoon Park [1], Chi-kyou Lee [1 *], Jong-Seok Moon [2 *]

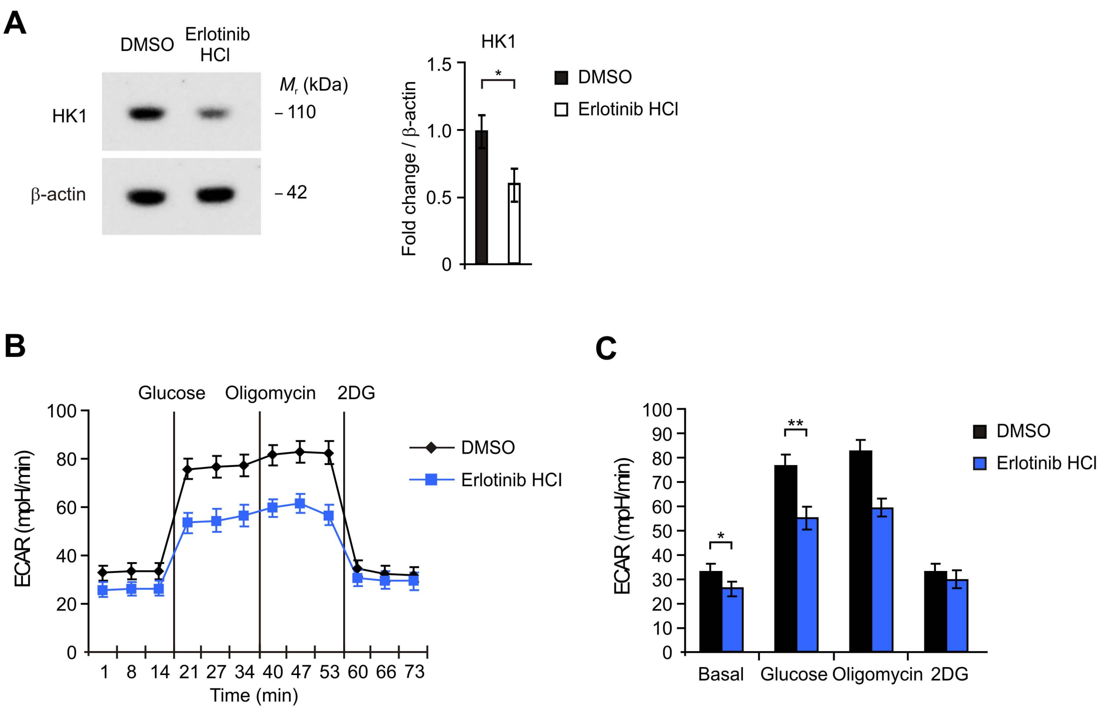

Supplemental Figure S1. Inhibition of EGFR suppresses HK1-dependent glycolysis in HEI-OC1 cells. (A) Representative immunoblot analysis for HK1 (right) and quantification for HK1 protein levels (left) from HEI-OC1 cells treated with Erlotinib Hcl (Erlotinib, 1 μM) or control (DMSO) for 4 h. For immunoblot, b-actin was used as loading control. Data representative of three independent experiments. Data are mean ±SEM. **P* < 0.05; by two-tailed student's t-test. (B) The levels of ECAR for glycolysis of glucose and (B) quantification of ECAR levels from HEI-OC1 cells treated with Erlotinib Hcl (Erlotinib, 1 μM) or control (DMSO) for 4 h. Data representative of three independent experiments. Data are mean ±SEM. **P* < 0.05; ** *P*<0.001 by two-tailed student's t-test.
